# The Role of Intelligence Quotient and Emotional Intelligence in Cognitive Control Processes

**DOI:** 10.3389/fpsyg.2015.01853

**Published:** 2015-12-01

**Authors:** Purificación Checa, Pablo Fernández-Berrocal

**Affiliations:** ^1^Department of Psychology, Faculty of Education Science, University of CádizCádiz, Spain; ^2^Department of Basic Psychology, Faculty of Psychology, University of MálagaMálaga, Spain

**Keywords:** interference suppression, impulsivity, Stroop, emotional intelligence, intelligence, cognitive abilities

## Abstract

The relationship between intelligence quotient (IQ) and cognitive control processes has been extensively established. Several studies have shown that IQ correlates with cognitive control abilities, such as interference suppression, as measured with experimental tasks like the Stroop and Flanker tasks. By contrast, there is a debate about the role of Emotional Intelligence (EI) in individuals' cognitive control abilities. The aim of this study is to examine the relation between IQ and EI, and cognitive control abilities evaluated by a typical laboratory control cognitive task, the Stroop task. Results show a negative correlation between IQ and the interference suppression index, the ability to inhibit processing of irrelevant information. However, the Managing Emotions dimension of EI measured by the Mayer-Salovey-Caruso Emotional Intelligence Test (MSCEIT), but not self-reported of EI, negatively correlates with the impulsivity index, the premature execution of the response. These results suggest that not only is IQ crucial, but also competences related to EI are essential to human cognitive control processes. Limitations and implications of these results are also discussed.

## Introduction

The relation between intelligence quotient (IQ) and cognitive control skills is well established (Blair, 2006; Shamosh and Gray, 2008). The IQ is commonly divided into two factors: fluid and crystallized intelligence. Fluid intelligence refers to the capacity to solve and think logically about novel problems. It is independent of the acquired knowledge. It is measured by a non-verbal test that requires abstract reasoning, such as a Matrices test. These tests are designed to reduce the influence of culture, educational level and verbal comprehension. On the other hand, crystallized intelligence depends on experience and knowledge and it could be defined as the ability to use these factors. Generally, Vocabulary and Verbal tests are used as a measure of this aspect of intelligence (Cattell and Raymond, 1963; Sternberg, 1999, 2005). Two important cognitive control abilities are filtering out interfering information and controlling impulsiveness. Interference suppression, i.e., filtering out interfering information, is a process that requires sustained attention in order to process relevant information and ignore irrelevant information. Furthermore, impulsivity could be considered as the consequence of dysfunctional inhibitory processes and strong impulses (premature execution of the response) and is modulated by dispositional and situational variables (Hofmann et al., 2009). These abilities are often measured by laboratory tasks such as the Stroop and Flanker tests. The common key to these tasks is that the participants must filter out interfering information as quickly as possible.

It is well-known that IQ, both fluid and crystallized intelligence is positively associated with some cognitive control processes (Detterman and Daniel, 1989; Duncan, 2000; Klingberg et al., 2005; Checa and Rueda, 2011; Duan and Shi, 2011; Rueda et al., 2012). The interference tasks require similar processes to be solved as those involved in the Matrices test of intelligence (fluid intelligence). Both tasks require processes such as representing information, attending to relevant information and inhibiting premature responses. Moreover, resolving interference tasks not only requires one to solve and think logically, but also it is an important ability to use previous experience and knowledge. The relation between IQ and cognitive control abilities could be suggesting that when resolving interference tasks, it is important to combine abstract reasoning (Matrices) and learned knowledge (Vocabulary). The association between IQ and cognitive control processes could be explained by assuming that crystallized intelligence may partially depend on fluid intelligence (Carroll, 1993), that is, a combination of both intelligences is important to resolve interference. However, the relation between impulsivity and IQ are more divergent. While some studies show a negative relation between impulsivity and IQ (Corr and Kumari, 1998; Lozano et al., 2014) others show that impulsivity is relatively independent of IQ (Plomin and Buss, 1973; Messer, 1976; Larsen, 1982).

Moreover, interference suppression and impulsivity have been related negatively to emotion regulation. Interference suppression has been associated with disruptive behavior and poor sociability in school (Checa et al., 2008), presence of externalizing and internalizing behavior problems (Olson et al., 2005; Valiente et al., 2007; Eisenberg et al., 2009). Impulsivity has also been conceptually and empirically linked to gratification delay, which requires the capacity to control impulses and postpone an immediate reward in order to obtain a larger reward (Mischel et al., 1989). Casey et al. (2011) showed that preschool children with less capacity to control impulses, as measured by delayed gratification tasks, display low self-control as adults 40 years later.

In contrast, there has been less research on the relation between Emotional Intelligence (EI) and performance of cognitive control tasks. EI constitutes another form of intelligence and the most widely applied theoretical models are mixed models and the ability model (Mayer et al., 2008). Mixed models conceptualize EI as a conglomeration of mental abilities and personality traits such as optimism, motivation, and stress tolerance (Mayer et al., 2008; Webb et al., 2013). The ability model, in contrast, defines EI as the integration of several capacities: “the ability to perceive accurately, appraise, and express emotion; the ability to access and/or generate feelings when they facilitate thought; the ability to understand emotion and emotional knowledge; and the ability to regulate emotions to promote emotional and intellectual growth” (Mayer and Salovey, 1997). In this research, we followed the EI ability model.

In recent years, there has been increasing interest in studying how these individual differences in EI affect cognitive skills and self-regulation. Emotionally intelligent people may use the capacity to adapt to others' strategy and context in order to attain their goals (Ford and Tamir, 2012). Some research has explored the influence of EI on cognitive processes, such as decision making and problem solving (Day and Carroll, 2004; Jordan and Troth, 2004; Reis et al., 2007; Demaree et al., 2010; Fernández-Berrocal et al., 2014; Webb et al., 2014). EI enhances the ability to learn and solve problems. People with a higher EI are able to generate a mood that allows them to do better on challenging cognitive tasks. Schutte and colleagues showed that participants with a higher self-reported EI resolved more cognitive tasks and did so better than those with a lower EI (Schutte et al., 2001). Also, the degree to which people focus on their feelings (Salovey et al., 1995), has also been associated with a better performed emotional Stroop task in general (Coffey et al., 2003). Additionally, and consistent with the idea that EI involves both crystallized and fluid components (Webb et al., 2013), the meta-analytic evidence indicates that ability EI using the Mayer-Salovey-Caruso Emotional Intelligent Test (MSCEIT) is positively correlated with verbal intelligence (0.26) and non-verbal intelligence (0.27; Kong, 2014).

EI is also important in self-regulated behavior, which can include impulse control. For instance, the EI ability to manage emotions using the MSCEIT is associated with aggression, and irresponsible behavior, such as alcohol and drug abuse (Riley and Schutte, 2003; Lomas et al., 2012; García-Sancho et al., 2014; Kopera et al., 2015). In recent years, a specific impulsive behavior has become extended in the population, the abuse of Smartphones and the Internet, and has also been related to low EI abilities (Billieux et al., 2008; Beranuy et al., 2009).

The aim of this study is to examine the relation between IQ and EI, and cognitive control abilities. It is novel to include a task-based measure of EI that allows more fine-grained investigation of the association between cognitive control and different aspects of EI. Three scores are derived from the Stroop task, including two interference indices (incongruent minus congruent trials) obtained for each participant, both in reaction times (RT) and errors (ER), and an impulsivity index, which is obtained by subtracting the mean RT for errors from the mean RT for correct responses. We explore the relation among the three cognitive control indices derived from the Stroop task, the IQ as measured by the Kaufman Brief Intelligence Test (K-BIT; Kaufman and Kaufman, 2000), and two commonly employed measures of EI following the ability model: the MSCEIT (Mayer et al., 2002; Extremera et al., 2006) and the Schutte Emotional Intelligence Scale (SEIS; Schutte et al., 1998). Many studies have shown that the process involved in resolving interference in a cognitive task is similar to the process involved in resolving the IQ test. For that reason, we expect that IQ will be related to performance in cognitive control tasks when the interference has to be resolved. Although many studies only examined the relation between cognitive control and fluid intelligence, we are interested in the relation between cognitive control and both crystallized and fluid intelligence. Like Carroll (1993), we think that crystallized intelligence (Vocabulary) may depend partly on fluid intelligence (Matrices), and that similar processes involved in resolving crystallized and fluid intelligence tasks are needed to resolve interference tasks. On other hand, there is evidence suggesting a positive relation between cognitive control and EI. We are interested in exploring the relation between EI measured by MSCEIT and two specific cognitive control indices: impulsivity and interference suppression. Moreover, there is data regarding the relation between cognitive control and emotional regulation. For that reason, we expect that both impulsivity and interference suppression, should also be negatively associated with self-regulation of emotions in the EI measures, such as Managing Emotions in the MSCEIT.

## Methods

### Procedure

Participants were tested at the Emotion Laboratory of the University of Málaga. Upon arrival, participants were informed of the general procedure of the sessions and given a few minutes to get comfortable in the laboratory setting before starting. The study involved two sessions. The first session took ~2 h, including time for instructions and breaks between questionnaires. Participants filled in the EI questionnaires (MSCEIT and SEIS) and completed the intelligence test (KBIT). In the second session, 1 week later, participants were verbally instructed on how to complete the experimental tasks (Stroop: described below). Task completion required about 1 h. The experimenter was present in the testing room throughout the sessions, but did not provide feedback to the participants apart from encouraging them to complete the task during breaks.

### Participants

Ninety-two undergraduate students from the University of Málaga participated in this study (73 women; mean age: 22 years; *SD* = 2.6 years). The age range was from 20 to 38 years. All participants came from Spain, and their first language was Spanish. The participants gave their written consent prior to participation. Participation in the study was voluntary.

The study was carried out in accordance with the Declaration of Helsinki. Ethics approval was obtained from the Research Ethics Committee, University of Málaga.

### Instruments

#### Kaufman brief intelligence test (KBIT; Kaufman and Kaufman, 2000)

The KBIT is an individually administered test with two subscales, Vocabulary and Matrices. The Vocabulary Scale is a measure of language and experience-related knowledge, and the Matrices Scale assesses abstract reasoning or fluid intelligence skills. The test provides scores for the two subscales as well as a composite IQ score. The Spanish version of this instrument has shown satisfactory psychometric properties (Cronbach alphas), Vocabulary α = 0.76, Matrices α = 0.82, and Composite IQ α = 0.83. In this study, we focused on the Vocabulary and Matrices Scales of the KBIT, and not on the total measure of KBIT, in order to know separately the relation between cognitive control and crystallized intelligence, the ability to use the previous knowledge (Vocabulary), and the relation between cognitive control and fluid intelligence, that includes abstract reasoning and problem solving in novel situations independently of experience (Matrices).

#### Mayer-salovey-caruso emotional intelligence test (MSCEIT v. 2.0; Mayer et al., 2002)

EI ability was measured using a Spanish translation of the MSCEIT that has similar psychometric properties to the original instrument (Extremera et al., 2006). This test has been validated for adults aged 17 years and older. The MSCEIT is not a self-reported measure. The MSCEIT uses two tasks to measure each of the four branches of EI (Perceiving, Facilitating, Understanding, and Managing Emotions), comprising a total of eight tasks. The instrument provides separate scores for each branch as well as an overall score for total EI; scores can be calculated based on expert or consensus norms. These two types of norms strongly correlate with each other (*r* > 0.90; Mayer et al., 2003), and the correlation between the two varies between 0.76 and 0.91 for each of the four branches separately (Mayer et al., 2003). In the present study, we used consensus norms to calculate scores for each of the four branches and for total EI. Scores computed by the test publishers are standardized (*M* = 100, *SD* = 15), and the split half reliability is 0.93, based on the consensus criterion.

#### Schutte emotional intelligence scale (SEIS; Schutte et al., 1998)

This scale is used to assess perceived EI. The SEIS is a self-report measure of EI consistent with the Salovey and Mayer (1990) model. The measure includes 33 items, which respondents rate on a five-point Likert scale (1 = *strongly disagree*; 5 = *strongly agree*). Prior work with the SEIS has found evidence of discriminant and criterion validity (Ciarrochi et al., 2002; Saklofske et al., 2003). We used the total score of the SEIS in this study. The Spanish version of this instrument has shown satisfactory psychometric properties (Ferrándiz et al., 2006). Cronbach's alpha in this study was 0.87.

#### Numerical stroop task

We used the numerical Stroop paradigm. Each trial started with a fixation point of 1500 ms duration. The target was presented until a response was given or for 800 ms, the target display consisted of two numbers. For half of the trials, the display was congruent, the numerically larger number was also physically larger (2, 6); and for the other half of the trial, it was incongruent, the numerically larger number was physically smaller (2, 6). The distance between the two numbers was two units, to control for the distance effect. The participants were required to indicate the numerically larger number. Thus, they had to press the left hand key “c” when the larger number was on the left or the right hand key “m” when the larger number was to the right. Following the response, a 500-ms feedback was provided. The feedback was a written word (“correct” for correct response; “error” for incorrect response; and “late” for omissions or off-time responses). After the feedback disappeared, the screen remained empty for a variable duration, randomly selected between 1000 and 1500 ms. Then the next trial began. Participants completed 336 trials divided into four blocks with small breaks between blocks. The dependent measures were RTs and the percentage of errors both in congruent and incongruent trials. We calculated two interference scores: interference in RT by subtracting the mean RT for incongruent trials from the mean RT for congruent trials, and interference in percentage of errors (% errors) by subtracting the percentage of errors for incongruent trials from the percentage of errors for congruent trials. We also obtained an index of impulsivity by subtracting the mean RT for error responses from the mean RT for correct responses.

## Results

Descriptive statistics on all measures are presented in Table 1.

**Table 1:** **Descriptive statistics of the measures**.

	**Mean**	**Min**	**Max**	***SD***
**STROOP TASK**				
Interference RT	54.3	17.0	95.5	19.5
Interference % errors	14.7	1.2	29.8	6.0
Impulsivity	82.5	2.0	42.6	8.3
**MSCEIT**				
Perceiving emotions	0.5	0.3	0.1	0.1
Facilitating emotions	0.4	0.3	0.5	0.0
Understanding emotions	0.5	0.4	0.6	0.0
Managing emotions	0.4	0.1	0.5	0.1
**SEIS**				
Total	4.0	2.4	4.7	0.4
**KBIT**				
Vocabulary	98.3	72	122	9.4
Matrices	98.7	72	116	7.9
Total	195.3	123	229	18

### Behavioral task: stroop

We conducted *t*-tests with RTs and percentage of errors as dependent measures when checking the effects of the Stroop task. For RT, results revealed a significant effect of condition type (congruent and incongruent), *t*_(91)_ = 26.6, *p* < 0.0001, *d* = 1.37, indicating faster responses in congruent (417 ms; *SD* = 33.2) compared to incongruent (472 ms; *SD* = 45.4) trials. Using the percentage of errors as dependent variables, we found a significant mean effect of condition type, *t*_(91)_ = 23.5, *p* < 0.0001, *d* = −2.83, indicating a smaller percentage of errors in congruent (2.9%; *SD* = 2.6) compared to incongruent trials (17.5%; *SD* = 6.8). The effect sizes for these analyses were found to exceed Cohen's convention for a larger effect (*d* = 0.80; Cohen, 1988).

We also examined differences in RT for correct compared to incorrect responses. Participants were faster when their responses were incorrect (351 ms; *SD* = 60) compared to correct (433 ms; *SD* = 94) responses, *t*_(91)_ = 9.5, *p* < 0.0001. The effect size was larger for this analysis (*d* = 1.05).

### Correlations

The correlation results are shown in Table 2. We have used the Cohen's effect size ranged from 0.10 (weak) to 0.50 (stronger). A correlation coefficient of 0.30 is considered a moderate correlation (Cohen, 1988). Correlations among the interference and impulsivity indices and the four MSCEIT branches and intelligence showed that impulsivity was negatively correlated with Managing Emotions, *r* = −0.23, *p* < 0.05, whereas interference (RT) was negatively correlated with Vocabulary, *r* = −0.25, *p* < 0.05, and Matrices, *r* = −0.24, *p* < 0.05. The Vocabulary measure of KBIT was also positively related to Facilitating Emotions, *r* = 0.22, *p* < 0.05, and Understanding Emotions, *r* = 0.28, *p* < 0.01. SEIS did not correlate with either interference or impulsivity scores.

**Table 2:** **Pearson correlation between Stroop task, emotional, and cognitive intelligences**.

	**1**	**2**	**3**	**4**	**5**	**6**	**7**	**8**	**9**	**10**
**STROOP**										
1. RT Interference										
2. Interference % errors	0.11									
3. Impulsivity	0.03	−0.12								
**MSCEIT**										
4. Perceiving emotions	−0.11	0.01	−0.06							
5. Facilitating emotions	0.11	0.07	−0.04	0.40^**^						
6. Understanding emotions	−0.06	−0.01	−0.13	0.22^*^	0.00					
7. Managing emotions	0.09	−0.04	−0.23^*^	0.12	0.18	0.05				
**SEIS**										
8. SEIS	0.07	0.13	−0.06	0.12	0.15	−0.11	0.14			
**KBIT**										
9. Vocabulary	−0.25^*^	−0.02	−0.12	0.15	0.22^*^	0.28^**^	−0.07	0.11		
10. Matrices	−0.24^*^	0.01	−0.01	−0.08	−0.06	0.14	−0.12	−0.01	0.42^**^	
11. Total	−0.27^**^	0.04	−0.04	−0.07	−0.05	0.19	−0.07	0.05	0.54^**^	0.68^**^

* p = 0.05.

** p = 0.01.

### Principle component factor analysis (PCA)

We conducted PCA using Varimax rotation with the data as exploratory analysis of the association found in the correlational analysis between EI and impulsivity and IQ and interference. Initially, the factorability was examined. The Kaiser-Meyer-Olkin measure of sampling adequacy was 0.58, although values between 0.5 and 0.7 are considered quite low (Hutchenson and Sofrionou, 1999), the commonly recommended value is 0.5. Bartlett's test of sphericity was significant [$\chi_{\left(10\right)}^{2}=60.04$, *p* < 0.001]. Two components were found: (a) Vocabulary and Matrices scores of the KBIT and the RT interference index, and (b) EI Managing Emotions and impulsivity index. The Vocabulary and Matrices scores of intelligence and the RT interference scores loaded highly on the first component (Eigenvalue = 1.66, explained variance = 33.36%), with rotated component loadings of 0.79 for Vocabulary, 0.77 for Matrices, and −0.61 for the RT interference. This component was interpreted as a cognitive component. The Managing Emotions and impulsivity scores loaded high on the second component (Eigenvalue = 1.24, explained variance = 24.74%) with rotated component loadings of 0.76 for Managing Emotions and −0.80 for impulsivity. This component was interpreted as the emotional component.

## Discussion

In the present study, we analyzed the relationship among IQ and EI, and cognitive control abilities using the interference and impulsivity indices obtained with Stroop tasks.

We observed that IQ, both Vocabulary and Matrices scores, correlated negatively with the interference index measured by the Stroop task, whereas Managing Emotions of the MSCEIT was related negatively to the impulsivity index. In this study the correlations ranged from *r* = 0.22 to *r* = 0.28, correlation coefficients indicated that a small/medium correlation exists. These coefficients are similar to those reported for other studies (Friedman et al., 2006; Billieux et al., 2008; Duan and Shi, 2011). Moreover, the PCA yielded two separate components: cognitive and emotional. Whereas, Vocabulary, Matrices and interference scores load on the first component, the second component is made up of impulsivity and Managing Emotions scores. Data from the correlations and from PCA both suggest an interesting association between the two types of intelligence and the two types of control processes. Our data suggests that impulsivity (premature execution of the response), but not interference (ability to inhibit processing of irrelevant information), is important for regulation of emotional information. However, interference, but not impulsivity, is related to IQ. These associations may not have a unique or simple explication.

One explication about the two relations between: (a) IQ: Vocabulary and Matrices scores and the interference suppression and (b) EI: Managing Emotions and the impulsivity could be found in the processes employed to carry out each one of these abilities. We used to think, as other authors claim (Dempster, 1991; Das, 2002), that interference or the ability to resist/suppress interference is an important component of intelligence. First, we found a relation between the intelligence scores of Matrices and the suppression of interfering information, like many other authors in the literature (Detterman and Daniel, 1989; Checa and Rueda, 2011; Duan and Shi, 2011; Rueda et al., 2012). The correlation between interference and Matrices ranged from *r* = 0.26 to *r* = 0.59. The first hallmark of fluid intelligence is abstract reasoning (Sternberg, 1999, 2005). We considered that interference, as a measure of cognitive control, is particularly important when it comes to solving tasks with a high load of reasoning, such as the task involved in the Matrices subscale of the KBIT. Solving information interference tasks requires representing information, attending to relevant elements and inhibiting task-irrelevant elements as well as other potentially distracting information. Although obviously, fluid intelligence is not reducible to interference, the mental process responsible for cognitive monitoring and control could be similar to that involved in the Matrices subscales. Using the Stroop test, we found divergent results. Whereas, Duan and Shi (2011) found that Stroop interference correlated with Raven's Progressive Matrices (*r* = −0.26), Friedman et al. (2006) and Benedek et al. (2014) failed to report a significant correlation between Raven's Progressive Matrices and Stroop interference. The inconsistent findings in the literature may be due to the dependent variables used in the Stroop tasks. Whereas, in the present study and in the studies of Checa and Rueda (2011) and Duan and Shi (2011), the interference index in the cognitive tasks was calculated as the RT for incongruent trials minus the RT for congruent trials, Friedman et al. (2006) calculated the interference index as the RT for incongruent trials minus the RT for neutral trials. The same measures of interference must be used to clarify this aspect in the future research. Many studies examining the relations between cognitive control and intelligence have focused on fluid intelligence and have largely ignored crystallized intelligence. We are also interested in examining the relation between crystallized intelligence (Vocabulary) and interference, as we expect that both Matrices and Vocabulary scores of intelligence are related to interference. In the literature reviewed, the correlation between Vocabulary and interference ranged from *r* = 0.16 to *r* = 0.35. For instance, Checa and Rueda (2011) showed that high Vocabulary scores are related to resolving interference in a Flanker task (*r* = −0.35). Friedman et al. (2006), using a naming Stroop task, found a correlation between interference and Vocabulary (*r* = 0.16). It could indicate that similar processes involved in resolving matrices and verbal/vocabulary test of intelligence are needed to resolve interference tasks. Our results also reveal a positive correlation between fluid (Matrices) and crystallized (Vocabulary) intelligence. It seems that crystallized intelligence may depend partly on fluid intelligence (Carroll, 1993). In contrast, we have not found a relation between impulsivity and both fluid and crystallized intelligence as other studies show (Corr and Kumari, 1998; Lozano et al., 2014). Our data is in line with some evidence that shows that impulsivity is relatively independent of IQ when impulsivity is measured with the reaction time tasks (Plomin and Buss, 1973; Messer, 1976; Larsen, 1982). Our measure of impulsivity seems to be more related to speedy response execution, while IQ seems to be related to information processing.

Previous findings have suggested a relationship between the MSCEIT and both fluid and crystallized intelligence (Webb et al., 2013; Kong, 2014). In our study, we found a positive correlation between the MSCEIT and Vocabulary scores of intelligence (KBIT), but not with Matrices scores. The current findings support a positive stronger association of EI with crystallized than with fluid intelligence. In any case, these associations between crystallized intelligence and EI should be considered with caution because the sample characteristics (e.g., high percentage of females) limit the possibility of generalizing the results. Regarding impulsivity, our data indicates that impulsivity correlates negatively with the Managing Emotions branch of the MSCEIT. Impulsivity could be seen as a response inhibition or a prevention of premature execution of the response: stopping or postponing a response (Nigg, 2001). In the present study, we found a negative correlation between the Managing Emotion measures of the MSCEIT and impulsivity. This data is congruent with studies from the cognitive literature showing that individuals who exhibited more impulsive behaviors displayed poor emotional control, using the same index of impulsivity as the one used in the present research (Pailing et al., 2002; Checa et al., 2014). Impulsivity has been related to emotion regulation even in early infancy. Children who showed short latency to grasp objects, or impulsivity, at six, ten, and 13 months also showed high anger frustration and aggression at age 7 years (Derryberry and Reed, 1996; Rothbart et al., 2000). The relation between impulsivity and emotion regulation seems to become stable over time. Impulsive children show low emotion regulation in their mid-forties (Casey et al., 2011). From the EI literature, there is evidence that EI is a determinant factor for impulse control. Lower EI, using self-reported measures, has been associated with behaviors reflecting a lack of control of impulsivity, such as drug, alcohol, Smartphone, and Internet abuse (Billieux et al., 2008; Beranuy et al., 2009). Using the MSCEIT, the relevance of managing emotions to deal with aggression or to regulate conflictive behaviors has been pointed out (Lomas et al., 2012; García-Sancho et al., 2014). It is important to note the relevance of the measure used in our study. Although the relation between emotional regulation and impulsivity has been found before, this is to our knowledge, the first testing of the relation using execution measures: impulsivity, as measured by a Stroop task, and emotion regulation, as measured by the Managing Emotions scale of the MSCEIT. In the EI literature, impulsive behaviors such as abuse of technology, alcohol, drugs, bullying, and aggression are usually assessed through questionnaires, but not through a behavioral performance task, such as the Stroop. We suggest that all measures used in this study based in the participant's execution reflect more objectively the processes implicated in each task examined. Self-report of EI seems to assume that participants can accurately assess and report their abilities. In contrast, the Managing Emotions of the MSCEIT is a measure based on participants' performance revealing EI abilities to resolve the task and emotional problems of the test. Some evidence shows that the MSCEIT measures EI abilities more accurately and reliably than self-report of EI (Brackett et al., 2006; Goldenberg et al., 2006; Webb et al., 2013; Cabello and Fernández-Berrocal, 2015). Moreover, when we used execution measures to evaluate IQ, EI and cognitive control processes in the same sample an interesting association appears between IQ and EI and the cognitive processes related to each other. This is exciting but only speculative and needs to be supported by future research using the same measures.

Evidence suggests a positive relation between EI in general and cognitive control (Day and Carroll, 2004; Jordan and Troth, 2004; Reis et al., 2007; Demaree et al., 2010; Fernández-Berrocal et al., 2014; Webb et al., 2014), but this study demonstrated that it is specifically a negative association between Managing Emotions and impulsivity. However, we did not find any association between self-reported measures of EI (SEIS) and impulsivity. It could be due to both Managing Emotions and impulsivity being measured based on participant's performance. Using the MSCEIT give us the opportunity to know which of the abilities of the EI could be associated with control. In the MSCEIT, the ability to regulate emotion is evaluated by the Managing Emotions ability. Moreover, the indices measured in the Stroop task target the processes involved in controlling the information (interference) or the response (impulsivity) in a controlled situation, in other words, the ability of the person to regulate or manage information and response. However, the rest of the EI abilities could be related to other cognitive processes. For example, we suggest that the ability of Perceiving Emotions could be related to some cognitive processes such as target detection.

In summary, data from our study provides evidence that people with higher IQs also resolve the interference Stroop tasks better. In the recent past, mounting evidence indicates that, although IQ is not reducible to interference, the ability to suppress interfering information is important to resolve tasks requiring abstract reasoning (IQ; Detterman and Daniel, 1989; Checa and Rueda, 2011; Duan and Shi, 2011; Rueda et al., 2012). Second, our data also shows a negative relation between EI abilities, specifically Managing Emotions, and impulsivity. Again, these data indicate that people who exhibit more emotion regulation are less impulsive when responding to cognitive tasks such as the Stroop task. We could consider this measure of impulsivity and Managing Emotions in EI as evaluating the same construct; emotional control that operates in “hot” situations but not reducible to each other. This negative relation between Managing Emotions in EI and impulsivity has to be replicated in future investigations using different samples.

## Limitations and future directions

The sample is small. This problem limits the future performance of further confirmatory analysis. The future studies have to replicate the results in a larger sample in order to generalize from a sample to the population. Concerning the Principal Component Analysis, it has to be seen as exploratory analysis in order to confirm the existence of two factors, which explain the 58% of variability. Moreover, inconsistency when measuring the same construct is a problem when comparing our results and the results shown in the literature. For example, we failed to find a relation between interference and emotional control although this relation has been found in the literature (Oldehinkel et al., 2004; Olson et al., 2005; Simonds et al., 2007; Checa et al., 2008). Also, self-reported EI in general or dimensions, such as Appraisal of Emotions or Emotional Attention (Austin, 2004), have also been related to performance in the Stroop task (Coffey et al., 2003). However, we found that interference is related to intelligence but not to emotion regulation as measured by the MSCEIT, as we expected. Previous research has provided support for the relation between self- or other-reported cognitive control, which includes interference suppression, and emotion regulation (Olson et al., 2005; Checa et al., 2008). We suggest that the discrepancy found could be due to the different methods used to evaluate both emotion regulation and cognitive control. The above-mentioned studies used self-reports or parents' reports of cognitive control, and they not only measured interference suppression, but also attention control (the capacity to control attention and to shift attention when desired) and activation control (the capacity to perform an action when there is a strong tendency to avoid it). Also, these cognitive ability measures are evaluated in the cited studies through a broad range of everyday situations, whereas our measure of interference is related to performance in the laboratory setting. Again, we recommend that future research use the same measures to assess cognitive control abilities, such as interference and impulsivity, and emotional abilities, such as Managing Emotions of the MSCEIT.

In summary, our data suggests that not only IQ is fundamental to human cognitive control processes, but also points out the significance of exploring the influence of EI.

### Conflict of interest statement

The authors declare that the research was conducted in the absence of any commercial or financial relationships that could be construed as a potential conflict of interest.
